# Anti-HTLV antibody profiling reveals an antibody signature for HTLV-I-Associated Myelopathy/Tropical Spastic Paraparesis (HAM/TSP)

**DOI:** 10.1186/1742-4690-5-96

**Published:** 2008-10-20

**Authors:** Peter D Burbelo, Elise Meoli, Hannah P Leahy, Jhanelle Graham, Karen Yao, Unsong Oh, John E Janik, Renaud Mahieux, Fatah Kashanchi, Michael J Iadarola, Steven Jacobson

**Affiliations:** 1 Neurobiology and Pain Therapeutics Section, Laboratory of Sensory Biology, National Institute of Dental and Craniofacial Research, Bethesda, MD 20892, USA; 2 Viral Immunology Section, Neuroimmunology Branch, National Institute of Neurological Disorders and Stroke, Bethesda, MD 20892, USA; 3 Metabolism Branch, National Cancer Institute National Institutes of Health, Bethesda, MD 20892, USA; 4 Unité d'Epidémiologie et Physiopathologie des Virus Oncogènes, CNRS URA 3015, Département de Virologie, Institut Pasteur, Paris, 75015, France; 5 The George Washington University Medical Center, Department of Microbiology, Immunology, and Tropical Medicine, Washington, DC 20037, USA

## Abstract

**Background:**

HTLV-I is the causal agent of adult T cell leukemia (ATLL) and HTLV-I-associated myelopathy/tropical spastic paraparesis (HAM/TSP). Biomarkers are needed to diagnose and/or predict patients who are at risk for HAM/TSP or ATLL. Therefore, we investigated using luciferase immunoprecipitation technology (LIPS) antibody responses to seven HTLV-I proteins in non-infected controls, asymptomatic HTLV-I-carriers, ATLL and HAM/TSP sera samples. Antibody profiles were correlated with viral load and examined in longitudinal samples.

**Results:**

Anti-GAG antibody titers detected by LIPS differentiated HTLV-infected subjects from uninfected controls with 100% sensitivity and 100% specificity, but did not differ between HTLV-I infected subgroups. However, anti-Env antibody titers were over 4-fold higher in HAM/TSP compared to both asymptomatic HTLV-I (*P *< 0.0001) and ATLL patients (*P *< 0.0005). Anti-Env antibody titers above 100,000 LU had 75% positive predictive value and 79% negative predictive value for identifying the HAM/TSP sub-type. Anti-Tax antibody titers were also higher (*P *< 0.0005) in the HAM/TSP compared to the asymptomatic HTLV-I carriers. Proviral load correlated with anti-Env antibodies in asymptomatic carriers (*R *= 0.76), but not in HAM/TSP.

**Conclusion:**

These studies indicate that anti-HTLV-I antibody responses detected by LIPS are useful for diagnosis and suggest that elevated anti-Env antibodies are a common feature found in HAM/TSP patients.

## Background

Human T lymphotropic virus type I (HTLV-I) is a retrovirus that infects 20 million people worldwide [1]. HTLV-I infection can cause a variety of human diseases including adult T-cell leukemia/lymphoma (ATLL) [2-4], HTLV-I associated myelopathy/Tropical Spastic Paraparesis (HAM/TSP) [5], infective dermatitis [6], and uveitis [7]. While the two major HTLV-I-associated diseases, ATLL and HAM/TSP, are present in all endemic areas, including Japan, the Caribbean basin, South America and parts of Africa, the incidence rates show geographic heterogeneity [1]. ATLL is an aggressive monoclonal proliferation of HTLV-1 infected CD4+ T cells that occurs mostly in adults. Perinatal HTLV-I infection is thought to be associated with a heightened risk of developing ATLL after a long latency period. Although the pathogenesis of ATLL is not completely understood, the HTLV-I regulatory protein Tax plays a critical role in cellular transformation by interfering with genome instability, cell cycle and apoptosis [8].

HAM/TSP is a chronic progressive neurodegenerative disorder that involves demyelination of the spinal cord and is characterized by CNS perivascular infiltration of inflammatory cells [9]. Epidemiological studies indicate that acquiring HTLV-I infection later in life through sexual contacts or through blood transfusion are linked to the future development of HAM/TSP a short time after infection. While HAM/TSP patients have high levels of anti-HTLV-I antibodies [10,11], lower anti-Tax antibodies are often found in ATLL patients, which may be due in part to Tax mutations that allow viral escape from cytotoxic T-lymphocyte (CTL) responses [12,13]. HAM/TSP patients show high HTLV-I proviral loads in peripheral blood lymphocytes [14-16] and increased spontaneous lymphoproliferation *in vitro *[17-19]. HAM/TSP patients also have high levels of HTLV-I-specific CTLs that have been reported to play an immunopathogenic role [20-22]. On the other hand, ATLL patients commonly exhibit immunodeficiency [23] and show ineffective anti-HTLV-I cell-mediated immunity [24].

Although there are adequate methods for determining if people are infected with HTLV-I, there are no serological diagnostic tests available for discriminating asymptomatic carriers from HAM/TSP patients or ATLL patients. Currently HTLV-I diagnosis is performed by immunoassays for HTLV-I gene products, HTLV-I-specific antibody production, detection of HTLV-I DNA, Southern blotting for ATLL diagnosis and more recently, proteomic approaches [25]. The ability to clearly distinguish between clinical outcomes of HTLV-I infections in a robust and simple serological test would have obvious clinical utility.

Currently, most immunoassays measuring anti-HTLV-I antibodies do not quantitatively evaluate multiple antigens and are incapable of detecting conformational epitopes in these antigens. We recently developed a highly sensitive immunoprecipitation technology called Luciferase Immunoprecipitation System (LIPS) that utilizes mammalian cell-produced, recombinant fusion protein antigens for efficiently evaluating antibody responses to multiple viral proteins and even a full virus proteome [26]. Here, LIPS was used to profile antibody responses to seven different HTLV-I proteins to gain a better understanding of the anti-HTLV-I antibody responses in non-infected controls, asymptomatic HTLV-I-carriers, HAM/TSP and ATLL sera samples. In addition to determining the prevalence of antibodies to these different proteins in HTLV-infected individuals, antibody titers were analyzed for correlations with HAM/TSP and ATLL clinical phenotypes, as well as proviral load.

## Results

### Diagnostically useful anti-Gag antibody titers are present in all HTLV-I infected individuals

Sera samples analyzed in this study were derived from 115 well-characterized participants including healthy volunteers, asymptomatic HTLV carriers, ATLL, and HAM/TSP patients. The gender, race/ethnic group and mean age of sample acquisition are summarized in Table 1.

**Table 1:** Characteristics of the participants used in the study

	Healthy Donor (n = 42)	ATL (n = 18)	Asymptomatic (n = 15)	HAM/TSP (n = 40)
Sex-no. (%)				
Male	26 (61.9%)	7 (38.9%)	4 (26.7%)	15 (37.5%)
Female	8 (19.0%)	11 (61.1%)	10 (66.7%)	25 (62.5%)
Unknown	8 (19.0%)		1 (6.7%)	

Race or Ethnic Group-no.				
White	20 (47.6%)	2 (11.1%)	5 (33.3%)	9 (22.5%)
African descent	10 (23.8%)	15 (83.3%)	4 (26.7%)	19 (47.5%)
Hispanic	3 (7.1%)	1 (5.6%)	5 (33.3%)	3 (7.5%)
Unknown	9 (21.4%)		1 (6.7%)	9 (22.5%)

Age at Sample Acquisition				
Mean (yr)	44	46	54	54

**Figure 1:**
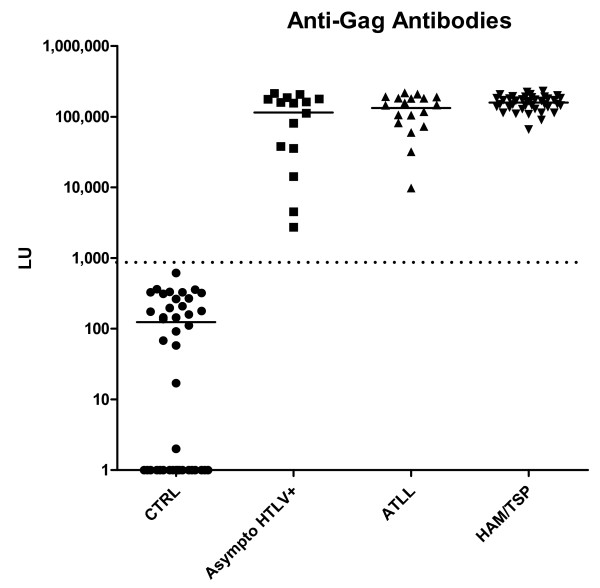
**LIPS detection of anti-HTLV-I Gag antibodies**. Each symbol represents individual samples from normal control, asymptomatic HTLV-infected, ATLL, and HAM/TSP patients. Antibody titers in LU are plotted on the Y-axis using a log_10 _scale. The dashed line represents the cut-off level for determining sensitivity and specificity for the particular antigen and is derived from the mean plus 5 SD of the antibody titer of the 42 normal volunteer samples. *P *values were calculated using the Mann Whitney U test. The solid line indicates the mean antibody titer per group.

While most previous studies evaluating anti-HTLV-I antibodies have used processed proteins of Gag such as p19 and p24, the full-length Gag was used in LIPS. Using the Cos1 cell containing fusion protein extracts, two independent measurements were made with 115 blinded sera in the LIPS format. From the average of these tests, the anti-Gag antibody titers showed values in the 115 sera ranging from 0 to 231,132 LU (Figure 1). The mean ± standard deviation (SD) of the anti-Gag antibody titer in the 42 normal HTLV-I seronegative controls was 123 ± 150 LU and it was significantly different (*P *< 0.0001) from the mean value of 143,250 ± 56,169 LU for the confirmed 73 HTLV-I infected samples including the asymptomatic carriers, ATLL and HAM/TSP samples. Using a value of 875 LU as a cut-off derived from the mean plus 5 SD of the controls revealed 100% sensitivity (73/73) and 100% specificity in detecting positive anti-Gag antibodies in the known HTLV-I positive samples. Some of weak background signals seen in control samples were below the cut-off value and were dramatically lower than any of the HTLV-I positive samples. While the anti-Gag antibody was highly useful for the diagnosis of HTLV-I infection, the mean anti-Gag antibody titers were not statistically different between the 15 asymptomatic HTLV-I carriers, 18 ATLL and 40 HAM/TSP HTLV-I positive patient groups (Figure 1).

### Anti-Env antibodies are markedly elevated in HAM/TSP compared to ATLL or asymptomatic HTLV-I-carriers

**Figure 2:**
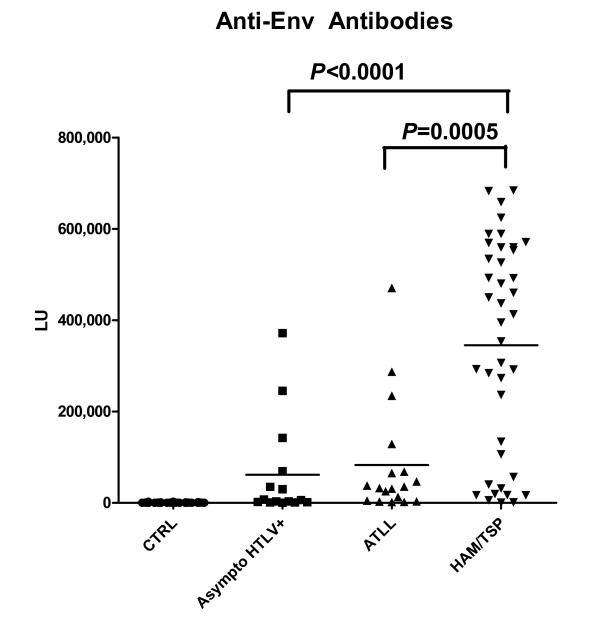
**LIPS detection of anti-Env antibodies**. Each symbol represents an average of two independent measurements from individual samples from normal control, asymptomatic HTLV-infected, ATLL, and HAM/TSP patients. The solid line indicates the mean antibody titer per group.

Anti-Env antibodies were also evaluated in the 115 sera samples (Figure 2). While the 42 normal HTLV-I seronegative control samples showed anti-Env antibody titers with a mean and SD of 730 ± 599 LU, the 73 known HTLV-I infected sera samples had a 300-fold higher mean and SD of 222,158 ± 232,681 LU. A Mann-Whitney U test showed a marked statistical difference in anti-Env antibody titers between the controls and HTLV-I infected patients (*P *< 0.0001). A cut-off of the mean plus 5 SD of the control subjects revealed 85% sensitivity (62/73) and 100% specificity in detecting positive anti-Env antibodies in the HTLV-I positive samples. The anti-Env antibody responses were less useful than the anti-GAG antibody titers to discriminate HTLV-I infected from uninfected controls and this is reflected by the slightly lower area under the curve (AUC) value of 0.95 for anti-Env antibodies verses 1.00 for the anti-Gag antibody test as determined by analyzing receiver-operating characteristics (ROC).

As shown in Figure 2, analysis of the mean anti-Env antibody titers in the different HTLV-infected groups revealed that the HAM/TSP patients had much higher serum antibody titers than the 18 ATLL or 15 asymptomatic HTLV-I-carriers. The mean anti-Env antibody titer in the HAM/TSP patients was 345,176 ± 232,983 LU, while the ATLL and asymptomatic HTLV-I-infected patients had mean titers of 82,825 ± 125,784 LU and 61,310 ± 109,976 LU, respectively. A Mann Whitney U test showed that the anti-Env antibody titers in the HAM/TSP patients were markedly different than the asymptomatic HTLV-I carriers (*P *< 0.0001) and ATLL patients (*P *< 0.0005). In contrast, the anti-Env antibody titers were not statistically different between the asymptomatic HTLV-I-infected and ATLL patients (*P *= 0.1782).

Inspection of patient characteristics did not reveal any obvious differences that explained the 4 ATLL and 4 asymptomatic HTLV-I-infected outliers with relatively high anti-Env antibody titers (Figure 2). For example, the patient characteristics (age, range 36–74; gender, one male and three females; race, one white and three African descent; or country of origin, one Jamaican; three U.S.) of the 4 asymptomatic HTLV-I-infected individuals showed no consistent pattern and none of these 4 individuals developed HAM/TSP during a 2–5 year follow-up. Furthermore, none of the ten HAM/TSP subjects with relatively low anti-Env antibody titers as a group significantly differed from the HAM/TSP cohort with respect to age, gender race or country of origin. However, all ten HAM/TSP patients were not classified as rapid progressors and three of the ten had a history of treatment with anti-retrovirals.

### LIPS analysis of anti-Tax antibodies

**Figure 3:**
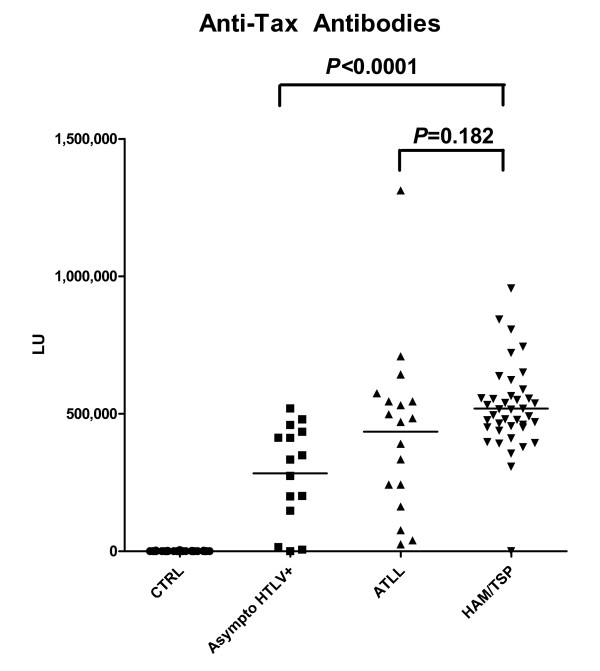
**Detection of positive anti-Tax antibodies**. Each symbol represents an individual sample from normal control, asymptomatic HTLV-infected, ATLL, or HAM/TSP patients. The solid line indicates the mean antibody titer per group.

HTLV-I Tax is a major regulator of gene expression in the human host and several studies have suggested anti-Tax antibodies are involved in the pathogenesis of HAM/TSP [27,28]. Using LIPS, the anti-Tax antibody response showed a wide dynamic titer range varying from 0 to 1.3 million LU in the entire sample set (Figure 3). The 42 normal HTLV-I seronegative control samples had a low mean anti-Tax antibody titer of 560 LU ± 826 LU. In contrast, the anti-Tax antibody titers for the 73 HTLV-I infected samples were almost 1000 times higher with a mean value of 449,609 ± 223,747 LU (*P *< 0.0001). Using a cut-off of the mean plus 5 SD of the control subjects revealed 85% sensitivity (14/15) in detecting positive anti-Tax antibodies in the HTLV-I asymptomatic carriers and 98% sensitivity (39/40) in detecting positive anti-Tax antibodies in the HAM/TSP samples. In the case of the ATLL samples, 100% (18/18) were found to have anti-Tax positive titers.

Inspection of the mean anti-Tax antibodies among the different HTLV-I infected groups showed that the 40 HAM/TSP patients had a mean anti-Tax antibody titer of 518,849 LU that was close to twice the mean value of 282,900 LU for the asymptomatic HTLV-I carriers. This elevated anti-Tax antibody in the HAM/TSP patients compared to the asymptomatic HTLV-I carriers was highly statistically significant (*P *< 0.0001). In contrast, no statistical difference (*P *= 0.1815) was found between the mean anti-Tax antibody titer of the HAM/TSP and ATLL samples.

### Few detectable antibody responses to other HTLV-I antigens

A previous study using LIPS to profile HIV-infected patients' humoral responses to the entire HIV proteome revealed antibody responses to all HIV proteins including the HIV reverse transcriptase and minor accessory proteins such as Rev and Vif [26]. To determine if LIPS could detect antibody responses to additional HTLV-I proteins, we tested for antibodies to HTLV-I encoded p12, p30, Rex and reverse transcriptase. While no detectable anti-p12, anti-p30 or anti-reverse transcriptase antibodies were detected (data not shown), several of the HTLV-I infected sera showed positive immunoreactivity towards Rex. Using a cut-off of 3,907 LU, the mean plus 5 SD of the anti-Rex antibody titers in the control samples, three HTLV-I positive sera were detected, comprised of two HAM/TSP samples with titers of 60,958 LU and 1.35 million LU and one ATLL sample with a titer of 92,044 LU. It should be noted that these anti-Rex antibody positive samples may represent high anti-HTLV-I antibody responding patients because all 3 of these samples were also positive for anti-Gag, anti-Env, and anti-Tax antibodies.

### Correlation of antibody titers with HTLV-I proviral load

**Figure 4:**
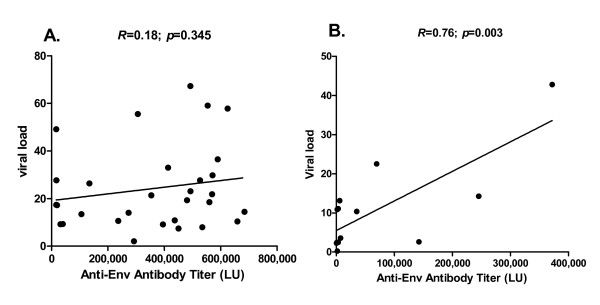
**Anti-Env antibody titer in asymptomatic HTLV-I-infected carriers correlates with the proviral load**. Asymptomatic HTLV-I-infected patients and HAM/TSP patients were evaluated for proviral load as described in the material and methods. (A.) Linear regression analysis showed that the anti-Env antibody titer determined by LIPS did not correlate in HAM/TSP patients. (B.) In contrast, proviral load significantly correlated (Pearson correlation *R *= 0.76) with anti-ENV antibody titers in asymptomatic HTLV-I carriers.

Recent studies indicate that higher HTLV-I proviral loads correlate with HAM/TSP [29,30]. In agreement with these studies, the mean HTLV-I proviral loads in the HAM/TSP patients were three-fold higher than the HTLV-I asymptomatic patients, which was statistically significant (*P *= 0.0067). Comparison by regression analysis of the HTLV-I proviral load with the HTLV-I antibody titer data revealed that there was no significant correlation between the different antibody titers determined by LIPS and the proviral load in the HAM/TSP patients. For example as shown in Figure 4A, the correlation between the anti-Env antibody titers and proviral load in HAM/TSP patients showed a Pearson correlation coefficient of only (*R *= 0.18, *P *= 0.345). In contrast, anti-Env antibody titers from asymptomatic HTLV-I carriers markedly correlated (Pearson *R *= 0.76; P = 0.003) with the HTLV-I proviral load (Figure 4B). Based on the covariance, the anti-Env antibodies explained 49% of the proviral load levels in the asymptomatic HTLV-I infected patients. In asymptomatic carriers, anti-Gag and anti-Tax antibody titers had no significant relationship with HTLV-I proviral load (data not shown). Together these results suggest that anti-Env antibody titers determined by LIPS may be a weak marker for the level of virus present in the blood of asymptomatic HTLV-I-infected patients, but not in HAM/TSP patients.

### HTLV-I antibody titers are relatively stable in longitudinal samples

**Figure 5:**
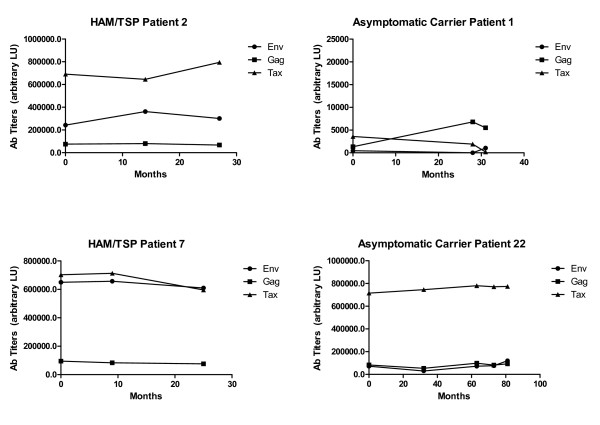
**Longitudinal analysis of HTLV-I anti-Env, Gag, and Tax antibody responses**. Data is plotted for two representative untreated HAM/TSP patients (left) and two HTLV-I asymptomatic carriers (right). Serum sampling dates are shown on the X axis and antibody titer data (LU) is on the Y-axis.

An additional set of blinded serial samples (n = 76) from a number of selected patients were also analyzed for anti-Gag, anti-Env, and-Tax antibodies. Representative results from several of these patients are shown in Figure 5, in which the anti-Gag, anti-Env, and anti-Tax antibody titers were relatively stable in both the asymptomatic HTLV-I carriers and HAM/TSP.

### Antibody profiles versus HTLV-I clinical phenotypes

Several of the HTLV-I antigens showed markedly higher antibody titers in HAM/TSP patients compared to ATLL and asymptomatic HTLV-I carriers. Therefore, ROC analysis was used to evaluate the most useful antibody markers. The anti-Env antibody titer showed the best discriminative power for identifying patients with HAM/TSP versus asymptomatic HTLV-I carriers and ATLL, as shown by the large AUC value of 0.83 from the ROC curve. Anti-Tax and anti-Gag antibodies were still informative but had lower AUC values of 0.72 and 0.61, respectively (data not shown). When a cutoff threshold greater than 100,000 LU was applied to the anti-Env antibody titers, the sensitivity and specificity for identifying HAM/TSP patients versus ATLL and asymptomatic HTLV-I carriers were 81% and 72%, respectively (Table 2). Using this 100,000 LU cut-off, the odds ratio for HAM/TSP versus ATL and asymptomatic was 11.1 (95% confidence interval 3.7–33.5). The presence of anti-Env antibody titers above 100,000 LU provides a fairly useful diagnosis (sensitivity 81%; specificity 72%; positive predictive value 75%) and shows the importance of monitoring these antibodies. For comparison, a higher cutoff value of greater than 200,000 LU yielded a sensitivity of 85% sensitivity and 70% specificity with an odds ratio of 13.1 (95% confidence interval 4.1–42.0). Additional analysis separately comparing the HAM/TSP patient with either asymptomatic HTLV-I carriers or ATLL yielded similar values (Table 2). For example, using the 100,000 LU cut-off of HAM/TSP versus asymptomatic patients yielded a sensitivity of 91% and 55% specificity with an odds ratio of 12.0 (95% confidence interval 2.8–51.4). Lastly, incorporation of anti-Tax and anti-Gag antibody titers into the HAM/TSP diagnostic algorithm did not significantly improve the sensitivity or specificity of the test (data not shown). This is because many of the samples positive for anti-Tax and anti-Gag antibodies were already positive for anti-Env antibodies and several additional false positives would have been detected in the asymptomatic HTLV-I carrier and ATLL groups.

**Table 2:** Sensitivity, specificity, positive and negative predictive values and odds ratio for the anti-Env antibody LIPS test in diagnosing HAM/TSP

Comparisons	Sensitivity	Specificity	PPV	NPV	OR (95% CI)
**> 100,000 LU**					

HAM/TSP vs ATLL & Asymp	81%	72%	75%	79%	11.1 (3.7–33.5)
HAM/TSP vs ATLL	88%	58%	75%	75%	10.5 (2.8–39.4)
HAM/TSP vs Asymp	91%	55%	75%	80%	12.0 (2.8–51.4)

**> 200,000 LU**					

HAM/TSP vs ATLL & Asymp	85%	70%	70%	85%	13.1 (4.1–42.0)
HAM/TSP vs ATLL	90%	56%	70%	83%	11.7 (2.8–47.9)
HAM/TSP vs Asymp	93%	52%	70%	87%	15.2 (3.0–77.9)

**Note**. PPV, positive predictive value; NPV, negative predictive value; OR, Odds ratio; CI, confidence interval.

## Discussion

LIPS technology provided quantitative measures of antibody titers to most of the proteins of HTLV-I. This simple modular system, expressing HTLV-I antigens as a series of Ruc fusion proteins followed by standardized chemiluminescent detection, efficiently evaluated patient humoral response to these different HTLV-I antigens. The substantial difference in anti-Gag antibody titers between the HTLV-I-infected samples and normal HTLV-I seronegative controls as determined by LIPS allowed for a universal and rigorous statistical cut-off (the mean of the 42 controls plus 5 standard deviations). Of the 7 antigens tested for diagnosing HTLV-I infection, the anti-Gag antibody test was the most informative, achieving 100% sensitivity and 100% specificity. The LIPS assay likely detects more conformational epitopes than alternative immunoassay formats providing high sensitivity, high specificity and robust signals that provide a substantial and clear distinction between positive and negative HTLV-I sera. Additionally, the high-throughput format used here makes this approach highly feasible for screening large numbers of sera samples.

From our LIPS studies profiling 7 different HTLV-I antigens, anti-Env antibody titers significantly correlated with the proviral load in asymptomatic HTLV-I seropositive samples (*R *= 0.76; *P *= .003). These results suggest that anti-Env antibody titers detected by LIPS may have utility in monitoring HTLV-I proviral load in asymptomatic individuals. This correlation between anti-ENV antibody titers and proviral load was unique to HTLV-I seropositive asymptomatic patients and consistent with previous studies demonstrating HTLV-I proviral load associations with anti-HTLV-I antibodies [12,13]. We now can suggest that in asymptomatic carriers, this correlation with HTLV-I proviral load was specific for antibodies to the Env region of HTLV-I. Of interest was the observation that anti-Env antibodies did not correlate with HTLV-I proviral loads in HAM/TSP patients. This may reflect higher levels of HTLV-I virus expression in patients with HAM/TSP reported to be associated with HTLV-I proviral integration sites that are not randomly distributed within the human genome but rather in transcriptionally active regions [31]. It has been proposed that an individual's steady state rate of HTLV-I proviral load and the accompanying risk of inflammatory diseases such as HAM/TSP, are the result of an equilibrium between HTLV-I replication and the host immune response [31]. In HAM/TSP, the immune response is fully engaged so that anti-Env antibody levels no longer correlate with HTLV-I proviral loads. In asymptomatic carriers, this saturation has not been achieved and HTLV-I proviral DNA levels highly correlate with antibody responses.

To date, most studies have found little utility in using antibody levels for monitoring disease progression or for sub-stratifying HTLV-I disease subtypes. Remarkably, in our study using LIPS, HAM/TSP patients had the highest level of anti-Env antibodies compared to ATLL or asymptomatic HTLV-I carriers. Anti-Tax antibodies were only elevated in the HAM/TSP patients compared to the asymptomatic HTLV-I carriers, but did not markedly differ with those of the ATLL patients. In contrast, anti-Gag antibodies were relatively similar between the different HTLV-I-infected patients. While antibody titers and proviral load have been reported to be higher in HTLV infected patients from Jamaica compared to Japan [32], simple demographics can not easily explain the differences in the sub-groups observed in this study. One implication of these findings is that anti-Env antibody titers above 100,000 LU have 75% positive predictive value and 80% negative predictive value for identifying the HAM/TSP sub-type (odds ratio, 12.0; 95% CI, 2.8–51.4). High anti-Env antibody titers were a better marker for HAM/TSP than anti-Tax antibodies, suggesting that the response to Env may have an important role in the progression to HAM/TSP. Alternatively, surface glycoproteins such as HTLV-I Env may be more immunogenic than intracellular Tax or Gag proteins. The detection of these high titer anti-Env antibodies in the HAM/TSP subgroup has not been previously reported and this is likely due to the fact other immunoassay formats such as Western blotting and ELISA poorly detect conformational epitopes and are not capable of quantitatively detecting these antibodies. Unlike published studies [33,34], we did not detect the lack of antibodies to Tax in ATLL versus asymptomatic carriers. This discrepancy may be due to the increased sensitivity in detecting anti-Tax antibodies in the LIPS format.

Previously, molecular mimicry against HTLV-I proteins was hypothesized to be responsible for HAM/TSP [27,28]. These studies showed that anti-Tax antibodies cross-reacted with a human protein, HNRNP-A1, associated with neurons. Based on the success of LIPS in detecting human autoantibodies associated with neurological diseases [35], the identification of human autoantigens (e.g. anti-HNRP-A1) associated with HAM/TSP may further improve the accuracy of this test and thereby potentially provide a non-invasive method to monitor and predict disease outcome in HTLV-I-infected patients. It is also likely that examining HTLV-I antibody and autoantibody titers in the CSF may provide additional information.

In summary, we have identified a predictive signature for HAM/TSP that would be economical and easy to implement in clinical laboratories. Currently we plan on investigating the contribution of anti-Env and anti-Tax antibodies in prospective studies of HTLV-I associated disease. Future studies using LIPS to discover additional auto-antibodies associated with clinical outcomes of HTLV-I infection may provide new tools for the prediction, diagnosis and monitoring of these disorders.

## Methods

### HTLV-infected patients and controls

The sera analyzed were derived from 115 well-characterized participants including healthy volunteers, asymptomatic HTLV-I carriers, ATLL (all subtypes were included with the majority of samples obtained from patients with the lymphomatous and acute leukemia subtypes) and HAM/TSP patients (diagnosed according to WHO guidelines) evaluated under Institutional Review Board-approved protocols of the NCI and NINDS, National Institutes of Health (Bethesda, MD). The gender, race/ethnic group and mean age of sample acquisition are summarized in Table 1. Additional longitudinal samples (n = 76) were also analyzed blindly from a subset of patients spanning a 2 year time period. Sera were kept at -80°C, aliquoted, then stored at 4°C, and measured as anonymous samples.

### Generation of Ruc-antigen fusion constructs

pREN2, a mammalian *Renilla *luciferase (Ruc) expression vector, was used to generate all plasmids [36]. HTLV-I cDNA clones for Gag, Env, reverse transcriptase, p30, p12, Rex and Tax were amplified by PCR with specific linker-primer adapters as described [26,36,37]. In each case, constructs employed full-length HTLV-I proteins fused to the carboxy terminus of Ruc. Additional Gag constructs representing the p24 and p19 processed proteins were also generated, but were found not to be as diagnostically useful as the full-length Gag. DNA sequencing was used to confirm the integrity of all the plasmid constructs. PCR primer sequences that were used to generate each construct are available on request.

### LIPS analysis

Following transfection of mammalian expression vectors, crude protein extracts were obtained as described [35]. For example, the full-length Gag construct yielded extracts with 1 × 10^9 ^light units (LU) of Ruc-Gag protein per 100 mm^2 ^plate of Cos1 cells (sufficient for ~300 serological tests). The LIPS immunoprecipitation assay was performed in a 96-well plate format at room temperature as described [37]. First, a "master plate" was constructed by diluting patient sera 1:10 in assay buffer A (20 mM Tris, pH 7.5, 150 mM NaCl, 5 mM MgCl_2_, 1% Triton X-100) in a 96-well polypropylene microtiter plate. For evaluating antibody titers by LIPS, 40 μl of buffer A, 10 μl of diluted human sera (1 μl equivalent), and 50 μl of the equivalent of 1 × 10^7 ^light units (LU) of Ruc-antigen Cos1 cell extract, diluted in buffer A, were added to each well of a polypropylene plate and incubated for 1 hour at room temperature. Next, 7 μl of a 30% suspension of Ultralink protein A/G beads (Pierce Biotechnology, Rockford, IL) in PBS were added to the bottom of each well of a 96-well filter HTS plate (Millipore, Bedford, MA). To this filter plate, the 100-μl antigen-antibody reaction mixture was transferred and incubated for 1 hour at room temperature on a rotary shaker. The washing steps of the retained protein A/G beads were performed on a BioMek FX work station (Beckman Coulter, Fullerton, CA) using an integrated vacuum manifold. After the final wash, LU were measured in a Berthold LB 960 Centro microplate luminometer (Berthold Technologies, Bad Wilbad, Germany) using coelenterazine substrate mix (Promega, Madison, WI). All LU data were obtained from the average of at least two independent experiments and corrected for background by subtracting the LU values of beads incubated with Cos1 cell extract, but without sera.

### HTLV-I proviral load

Real-time PCR analysis of HTLV-I (Tax) proviral load was performed as previously described [38]. DNA was extracted from 1 × 10^6 ^cells using Puregene DNA Isolation Kit (Gentra, Minneapolis, Minnesota, United States), and 100 ng of the sample DNA solution was analyzed by this system. The HTLV-I proviral DNA load was calculated by the following formula: copy number of HTLV-I (pX) per 100 cells = (copy number of pX)/(copy number of β-actin/2) × 100.

### Data analysis

The GraphPad Prism software (San Diego, CA) was used for other statistical analysis, including evaluating test performance by area under the curve (AUC). Results for qualitative antibody titers between the controls, seroindeterminates, asymptomatic HTLV-I carriers, HAM/TSP and ATLL are reported as the mean ± SD. Mann-Whitney U tests were used for comparison of antibody titers in different groups. Statistical significance of the regression analysis was evaluated by Pearson correlation coefficient and the level of significance was set at *P *< 0.05. For the calculation of sensitivity and specificity between HTLV-I infected and uninfected samples, the cut-off limit for each antigen was derived from the mean value of the 25 control samples plus 5 standard deviations. The sensitivity, specificity, negative predictive value, positive predictive value and diagnostic odds ratio were calculated by using 2 × 2 contingency tables and calculated using the GraphPad software. The multivariate data which included antibody titers and clinical covariates was also evaluated using the RapidMiner  suite of data mining tools.

## Competing interests

The authors declare that they have no competing interests.

## Authors' contributions

FK initially conceived of the study. FK and RM provided a pilot set of sera for proof of concept. PB generated the needed constructs. PB and HL analyzed the sera by LIPS. SJ, EM, JG, KY, UO, and JJ provided the sera samples used in this study, participated in the design of the experiments and analyzed the data. PB analyzed the data and drafted the manuscript. MI funded the study. All authors read and approved the manuscript.
